# Experimental infection of Asian house geckos with *Enterococcus lacertideformus* demonstrates multiple disease transmission routes and the *in-vivo* efficacy of antibiotics

**DOI:** 10.1038/s41598-021-92999-y

**Published:** 2021-07-05

**Authors:** Jessica E. Agius, Karrie Rose, Jon-Paul Emery, David N. Phalen

**Affiliations:** 1 Faculty of Science, Sydney School of Veterinary Science, University of Sydney, J.L. Shute Building C01A, 425 Werombi Road, Camden, NSW 2570 Australia; 2 Australian Registry of Wildlife Health, Taronga Conservation Society Australia, Mosman, NSW 2088 Australia; 3 Faculty of Science, School of Biological Sciences, University of Western Australia, Stirling Highway, Crawley, WA 6009 Australia; 4 Texas A&M School of Veterinary and Biomedical Sciences, Schubot Exotic Bird Health Center, College Station, Texas, 75189 USA

**Keywords:** Herpetology, Antibiotics, Biofilms, Bacterial genes, Bacterial infection, Inflammation

## Abstract

The disease caused by *Enterococcus lacertideformus* is multisystemic and ultimately fatal. Since its emergence, the bacterium has significantly impacted the captive breeding programs of the extinct in the wild Christmas Island Lister’s gecko (*Lepidodactylus listeri*) and blue-tailed skink (*Cryptoblepharus egeriae*). The bacterium’s pathogenicity, inability to grow *in-vitro*, and occurrence beyond the confines of Christmas Island necessitated the development of an experimental infection and treatment model. Asian house geckos (*Hemidactylus frenatus*) were challenged with a single dose of *E. lacertideformus* inoculum either by mouth, application to mucosal abrasion or skin laceration, subcutaneous injection, coelomic injection, or via co-housing with an infected gecko. Five healthy geckos acted as controls. Each transmission route resulted in disease in at least 40% (n = 2) geckos, expanding to 100% (n = 5) when *E. lacertideformus* was applied to skin laceration and mucosal abrasion groups. Incubation periods post-infection ranged between 54 and 102 days. To determine the efficacy of antibiotic treatment, infected geckos were divided into six groups (enrofloxacin 10 mg/kg, per os (PO), every 24 h (q24), amoxicillin-clavulanic acid 10 mg/kg, PO, q24, enrofloxacin 10 mg/kg combined with amoxicillin-clavulanic acid 10 mg/kg, PO, q24, rifampicin 15 mg/kg, PO, q24, clarithromycin 15 mg/kg, PO, q24, and untreated controls) for 21 days. Response to treatment was assessed by the change in lesion size, bacterial dissemination*,* and histological evidence of a host immune response. Irrespective of the antibiotic given, histology revealed that geckos inoculated by skin laceration were observed to have more extensive disease spread throughout the animal’s body compared to other inoculation routes. The reduction in the average surface area of gross lesions was 83.6% for geckos treated with enrofloxacin, followed by the combination therapy amoxicillin-clavulanic acid and enrofloxacin (62.4%), amoxicillin-clavulanic acid (58.2%), rifampicin (45.5%), and clarithromycin (26.5%). Lesions in geckos untreated with antibiotics increased in size between 100 and 300%. In summary, enrofloxacin and amoxicillin-clavulanic acid show promising properties for the treatment of *E. lacertideformus* infection in geckos. The Asian house gecko *E. lacertideformus* infection model therefore provides foundational findings for the development of effective therapeutic treatment protocols aimed at conserving the health of infected and at-risk reptiles.

## Introduction

Lister’s geckos (*Lepidodactylus listeri*) and blue-tailed skinks (*Cryptoblepharus egeriae*), once abundant on Christmas Island, are now extinct in the wild^1^. These critically endangered lizards are maintained only in conservation breeding facilities on Christmas Island and at Taronga Zoo, Sydney, Australia. The breeding programs for both species on Christmas Island are threatened by a recently emerged bacterium, *Enterococcus lacertideformus,* which has caused two outbreaks in the Christmas Island captive breeding facility. The initial outbreak of *E. lacertideformus* led to deaths of more than 40 Lister’s geckos and ten blue-tailed skinks in the breeding enclosures. The subsequent outbreak of the disease occurred in partially enclosed outdoor exclosures housing the blue-tailed skink male breeding stock, resulting in the deaths of more than 30 individuals. Both outbreaks of the disease were likely initiated by direct contact with infected free-ranging invasive reptiles, and the outbreaks were ultimately controlled by depopulation of affected and in contact lizards. Treatment was not considered an option at the time because the susceptibility of *E. lacertideformus* to antibiotics was not known^2^.

Untreated disease caused by *E. lacertideformus* is uniformly fatal. Animals infected with *E. lacertideformus* initially exhibit swellings composed of a subcutaneous white gelatinous material predominately localised to the face that subsequently disseminates, forming often coalescing nodules in multiple organ systems. Microscopically the lesions are composed of bacteria that are suspended in a thick biofilm. Bacterial aggregates grow by expansion, replacing the surrounding normal tissue and causing bone lysis. In most instances the lesions are not accompanied by inflammation, although uncommonly, aggregates of lymphocytes are found in the tissues adjacent to the lesions. The course of the disease is slow but progressive, with infected lizards surviving three weeks to four months after the initial lesions are observed^2^. How *E. lacertideformus* is acquired is not known. However, given that the initial lesions develop on the face, it is possible that infection occurs through bite wounds from other lizards or following colonisation of the oral cavity from an environmental source. To date, *E. lacertideformus* has not successfully been cultivated *in-vitro* using traditional bacterial isolation techniques, embryonated chicken eggs, and reptile cell lines^2^.

*Enterococcus lacertideformus* is enzootic in wild reptiles on Christmas Island. Surveys of the free-ranging invasive mute (*Gehyra mutilata*) and Asian house geckos (*Hemidactylus frenatus*) found animals infected with *E. lacertideformus* at multiple sites across the island over a period of four years^3^. Thus, the organism poses a continued threat to both the captive breeding program for Lister’s geckos and blue-tailed skinks on Christmas Island and any effort to release these species back into the wild.

It is also likely that *E. lacertideformus* is not confined to Christmas Island and threatens other species of reptiles. A morphologically identical bacterium causing indistinguishable gross and microscopic lesions was described in Singapore house geckos (*Gekko monarchus*) in Asia^4^, and in five species of lizards from Europe^5^. Both studies ascribed the agent to the *Streptococcus* genus of bacteria, however, at the time of publication, enterococci were classified within that genus. Efforts to culture the organisms were unsuccessful^4,5^. More recently, free-ranging brown anoles (*Anolis sagrei*) in Florida, United States of America were also observed with morphologically identical facial and multisystemic microscopic lesions. Amplification of a 1400 bp segment of the 16s rDNA gene from DNA extracted from these lesions revealed that it was 100% identical to *E. lacertideformus*^6^.

Given the uniformly fatal nature of infection and the vulnerability of insular reptile species, disease mitigation strategies are required. Therefore, understanding the modes of transmission, clinical course of infection, and susceptibility of *E. lacertideformus* to antimicrobial treatments are critically important to inform disease control and management. Based on a recent metagenomics study^7^, it appears that *E. lacertideformus* has limited antibiotic resistance and is likely susceptible to fluoroquinolones, macrolides, broad-spectrum penicillins, and rifamycins, antimicrobials that are often effective against other enterococci and have the added advantage of high penetrating capacity of biofilms^8,9^. These antibiotics have also been shown to be safe when administered to reptiles and can be given orally, which is a significant advantage when treating reptiles as small as two grams. Additionally, single-administration pharmacokinetic trials using orally administered enrofloxacin in the Asian house gecko showed that with appropriate dosages, therapeutic plasma concentrations can be achieved. However, no specific antibiotic protocols have been developed for Asian house geckos, or the Christmas Island endemic Lister’s geckos and blue-tailed skinks.

The aims of this study are twofold. The first is to identify a reproducible experimental model of infection that can shed light on the epizootiology and disease dynamics of this bacterium. The second aim is to assess the efficacy and practicality of five antibiotic treatment protocols that could be used to treat individual reptiles or captive populations of reptiles infected with *E. lacertideformus*.

## Materials and methods

### Animal ethics

The research protocol and use of wild reptiles were approved by the University of Sydney Animal Ethics Committee (AEC) (2018/1380) on 16 July 2018 in compliance with the NSW Animal Research Act 1985, and the Australian code for the care and use of animals for scientific purposes. The authors complied with the ARRIVE guidelines 2.0 for the reporting of animal research conducted in this study^10^.

### Acquisition of experimental geckos

Asian house geckos were collected from a location on Christmas Island (10°28′20.4″ S 105°34′44.2″ E) where infected geckos have never been observed. The geckos were physically inspected for lesions characteristic of *E. lacertideformus* infection and body condition scored (range 1 (poor) to 5 (well-muscled)) . Only geckos that appeared healthy and had a body condition score of three or greater were used. Geckos that did not meet these criteria were euthanised. Thirty-five mature geckos (18 males, 17 females) were randomly assigned to seven treatment groups of five animals using the random integer generator *numpy.random.randint* in Python v3.8^11^, and acclimatised for ten days. A sample size of five per treatment group was determined to be sufficient to detect a route of infection that resulted in a 50% infection rate using the formula: n = [1 − (1 − P1)^1/d^] [N − ((d − 1)/2)], where n is the sample size, P is the probability of detecting at least one case of the disease if it is present in the population [0.95], N is the population size [10,000], and d is the number of detectable cases in the population; where d = population size x prevalence x sensitivity of the test [10,000 × 0.5 × 0.95]^12^.

Five naturally infected Asian house geckos with lesions characteristic of *E. lacertideformus* were captured from a location on Christmas Island (10°25′55.7"S 105°40′13.4"E) known to have a high prevalence of *E. lacertideformus* infection. Three of the five affected geckos were euthanised and their tissues harvested to prepare the inoculum, while the remaining two geckos (1 male, 1 female) were assigned to the infection by contact trial. An additional ten Asian house geckos naturally infected with *E. lacertideformus* were captured at the same location and used in the treatment trial.

### Experimental infection model

#### Housing and husbandry

Geckos were individually housed in PenPal terrariums (30.0 cm × 18.0 cm × 18.0 cm) (Living World PenPals). Only geckos allocated to the ‘infection by contact’ treatment were housed as pairs. Each terrarium contained a large (18.0 cm × 3.5 cm) and small (15.0 cm × 2.0 cm) hide made of PVC pipe. Artificial foliage (15.0 cm × 10.0 cm) was provided as an additional form of refuge, and water was refreshed daily. Geckos were misted with water twice weekly and fed three days per week a mixture of live termites, moths, and crickets. All housing was contained within an escape-proof facility where the ambient temperature ranged from 26 to 30 °C.

#### Infection trials

Oral and skin swabs (Dacron, AMSL Scientific) were collected from all presumed disease-free and naturally diseased geckos prior to inoculation to confirm the absence or presence of *E. lacertideformus* infection via a real-time (qPCR) assay.

A fine needle aspirate (FNA) was collected from the lesions of five naturally infected geckos and subjected to cytology (Gram-positive cocci in chains encapsulated by a thick, lightly staining mucoid matrix), and qPCR to confirm *E. lacertideformus* infection. Diseased tissues from the head and oral mucosa were aseptically collected and homogenised in phosphate buffered saline (PBS) (pH 7.5 ± 0.5; Sigma-Aldrich). The homogenised solution was serially diluted to achieve a concentration of approximately 4 × 10^6^ organisms/mL. All experimentally infected geckos were inoculated with 0.02 mL (~ 80,000 organisms).

The infection trial included six routes of inoculum delivery (Table 1). Geckos in the control, oral cavity, and subcutaneous groups were inoculated using manual restraint. For mucosal abrasion, skin laceration, and coelomic cavity challenges, geckos were immobilised with a subcutaneous injection of Alfaxalone (5 mg/kg, Alfaxan, Jurox Animal Health) prior to inoculation. Following inoculation, all geckos were immediately returned to their enclosures. Sedated geckos were monitored for 20 min to ensure adequate recovery.

**Table 1:** The treatment groups used in the experimental infection trial.

Treatment	Route	Bacterial dose*	Inoculum volume (mL)	Site of Inoculation
Control	SC	0	0.02	Saline injection into the loose skin 2 mm off the ventral midline, and 5 mm cranial to the pelvis
Oral cavity	PO	80,000	0.02	Direct administration into the oral cavity
Subcutaneous	SC	80,000	0.02	Subcutaneous injection into the loose skin at the medial aspect of the neck
Mucosal abrasion		80,000	0.02	Shallow abrasion of the gingiva on the right side of the face using a cotton swab. Inoculum applied to abraded surface
Skin laceration		80,000	0.02	Shallow skin laceration (1 mm deep and 5 mm long) on the right lateral side of the face rostral to the ear ostium. Inoculum applied to wound surface. Wounds covered by a light-weight adhesive bandage for 24 h
Coelomic cavity	IC	80,000	0.02	Co-housed with an infected animal for ten days
Co-housing		n/a	n/a	Natural infection means

*SC* subcutaneous; *PO* per os; *IC* intracoelomic.

*Total number of organisms administered per injection.

All experimentally challenged geckos were held for a maximum of four months or until characteristic signs of *E. lacertideformus* developed, whichever occurred first. Oral swabs, and FNAs expressed onto swabs were collected from geckos that developed gross lesions (lesions visible to the naked eye) to confirm *E. lacertideformus* infection by qPCR, in addition to cytology of lesions. Infected geckos were enlisted into the antibiotic treatment trial. Four geckos that developed lesions were untreated, held for 28 days, and euthanised, constituting an untreated control group.

During the infection by contact phase of the infection trial, two affected geckos (a male and a female) were initially individually co-housed for ten days with a disease-free gecko of the opposite sex. The two affected geckos were then co-housed with a single disease-free gecko of the same sex for ten days. The affected male was then exposed to a third gecko; a disease-free male for ten days. Following trial completion, both affected geckos were euthanised and diseased tissues collected to confirm the lesions were caused by *E. lacertideformus*. All five geckos exposed to the infected geckos were held for a period of four months.

#### Monitoring

All geckos in the infection trial were observed daily in their enclosures, co-housed geckos were monitored for bite wounds or other evidence of aggressive behaviour. Twice weekly the lizards were weighed and visually inspected. Body condition score, food consumption, and faecal production were recorded, in addition to any observations that might relate to their health or disease status. When animals developed lesions characteristic of *E. lacertideformus* disease (e.g. facial swelling, epidermal nodules, and mass formation in the coelomic cavity or within viscera as determined by transillumination), the progression of their lesions were measured and photographed.

#### Endpoint and euthanasia

Euthanasia of all experimental geckos were undertaken if animals met the euthanasia end-point criteria, which included (1) a reduction of body condition score to two or less, or (2) facial or other swellings that interfered with normal activity, or (3) decreased appetite or anorexia. Geckos were euthanised with a subcutaneous injection of alfaxalone resulting in an overdose of the anaesthetic agent, and decapitated.

### Experimental treatment trial

Geckos that developed *E. lacertideformus* disease (confirmed by cytology and qPCR) during the infection trial (n = 9), and wild-caught geckos naturally infected (n = 10) were randomly allocated to the five antibiotic treatment groups using the random integer generator *numpy.random.randint* in Python v3.8^11^. The five treatments consisted of: enrofloxacin, 10 mg/kg, per os (PO), every 24 h (q24); rifampicin, 15 mg/kg, PO, q24; clarithromycin, 15 mg/kg, PO, q24; amoxicillin-clavulanic acid 10 mg/kg, PO, q24; and a combination of enrofloxacin, 10 mg/kg and amoxicillin-clavulanic acid 10 mg/kg, PO, q24. Four geckos were allocated into each treatment, with the exception of the combined therapy (enrofloxacin and amoxicillin-clavulanic acid) group that contained only three geckos due to a limited number of available animals. Treatment was administered seven days after the first signs of *E. lacertideformus* were observed. Geckos were treated for a total of 21 days and then euthanised. Some animals were euthanised earlier if end-point criteria were reached. All animals in the treatment trial were monitored as described for the infection trial. Lesions were described, photographed, and measured twice weekly on their longitudinal and transverse axes. As a means of assessing antibiotic efficacy, the percentage change of the lesion was calculated for each gecko by comparing the surface area of the lesions at day 7 after signs were observed (day 1 of antibiotic treatment) and at euthanasia (day 21 of antibiotic treatment). For each gecko, the total surface area of the lesion(s) was determined before and after treatment. The change in lesion surface area was determined by the formula: 100% × (A2 − A1)/A1, where A2 was the surface area after treatment and A1 was the surface area before treatment. The percent change was then grouped for each antibiotic treatment and no treatment. A boxplot was generated to compare the percent change in the lesion size across treatments using the pandas library v.1.2.0^13^ in Python v3.8^11^.

### Sample collection and processing

During post-mortem examination, oral swabs, a single liver lobe (as disease spread to the liver was common in naturally infected lizards), and a mid-sagittal section of the head were taken and stored in 100% ethanol for qPCR analysis. Tissues with suspected *E. lacertideformus* lesions were also collected and stored in RNA-later (Sigma-Aldrich) and frozen for qPCR analysis. The remains were fixed in 10% neutral buffered formalin for histological examination.

Prior to DNA extraction, oral swabs (n = 107), skin swabs (n = 45), lesion FNA swabs (n = 22), and tissues (n = 50) were rehydrated with four PBS washes to remove residual fixative. Tissues were mechanically ground and digested with proteinase K for 3 h. Pure genomic DNA was extracted from swabs using the buccal swab protocol from the QIAamp DNA mini extraction kit (Qiagen), and DNA was extracted from suspected *E. lacertideformus* diseased tissues using the animal tissue protocol from the DNeasy Blood and Tissue Kit (Qiagen).

### Quantitative PCR (qPCR) development and validation

#### qPCR primers, probes, controls, and conditions

The National Center for Biotechnology Information (NCBI) Primer-BLAST Tool^14^ was used to design a primer and probe set specific to a short fragment of the *E. lacertideformus* glucose-6-phosphate dehydrogenase (gdh) house-keeping gene (Table 2)*.* Oligonucleotides and probe were synthesised by Integrated DNA Technologies (IDT, USA).

**Table 2:** Primer and probe set used in the *E. lacertideformus*-specific qPCR assay.

Sequence ID*	5′ → 3′ sequence	Length (bp)	Tm (°C)	GC (%)	Amplicon Length (bp)
*EntL—Forward*	CCAAATAATAGATGCGATTCCC	22	59	40.9	171
*EntL—Reverse*	CTACTATCCAGTCACTCAATCC	22	59	45.5	
*EntL—Probe*	TGGGTTGAATCATTGACATCGTGAGA	26	66	42	

Primers were optimised by testing forward and reverse primer concentration combinations of 150, 300, 600, and 900 nM with a fixed probe concentration of 250 nM. The primer:probe concentration that was most efficient, yielding the lowest quantification cycle (Cq), lowest variation in replicates, and negative no template control (NTC) was chosen. Primer annealing temperatures were optimised stepwise by increasing the annealing temperature from 54 to 64 °C in increments of 2 °C. The annealing temperature with the lowest Cq, highest reproducibility between replicates, detection of the target DNA, and a negative NTC was chosen.

#### qPCR specificity

The specificity of the oligonucleotides was confirmed by scanning them against the NCBI GenBank Database using BLAST^15,16^. A DNA panel of non-target bacterial species (including near relatives of *E. lacertideformus*) was used to validate the specificity of the qPCR assay. The non-target controls were *Enterococcus villorum* F1129D*, Enterococcus faecium* AUS0085, *Enterococcus faecalis* ATCC 29212*, Staphylococcus aureus* NCTC 6571*, Escherichia coli* NCTC 10418, and *Pseudomonas aeruginosa* ATCC 27853. Additional non-target isolates included vancomycin-resistant *Enterococcus faecium, Enterococcus durans, Enterococcus hirae, Enterococcus villorum, Streptococcus anginosus, Streptococcus* Group F, and *Streptococcus* Group G that were all isolated at the Taronga Zoo Clinical Pathology Laboratory.

#### qPCR efficiency, reproducibility, and limit of detection

A double stranded 499 bp artificial gBlock fragment was designed and synthesised by Integrated DNA Technologies (IDT, USA) to determine the limit of detection (LOD); the absolute minimum number of copies detectable by the assay.

The LOD was determined using a tenfold dilution series of the gBlock from 1.22 × 10^8^ to 1.22 gene copies/µL. Each dilution series was run in five replicates. The lowest concentration of gBlock that produced a Cq value in all five replicates was considered the LOD. Quantification cycle values over 38 were removed and classed as non-detectable. Amplification efficiency was determined by plotting the Cq values versus the gBlock dilution and calculating the linear slope. The coefficient of determination (*R*^2^) was also calculated from this data.

### Enterococcus lacertideformus detection

#### qPCR diagnostic assay

DNA extracted from alcohol-fixed swabs, FNAs, and tissue samples were screened using the *E. lacertideformus*-specific qPCR assay. The following qPCR thermocycling conditions were used: 95 °C for 3 min, 40 cycles of 95 °C for 10 s, and 56 °C for 40 s. Each 10 µL reaction consisted of 5 µL of SensiFAST Probe No-ROX Kit (Bioline), 2 µL of DNA (5 ng), 0.6 µL of forward and reverse primers (10 pmol/µL), 0.25 µL probe (10 pmol/µL), and 2.15 µL PCR water. A NTC (PCR water) and positive control (confirmed *E. lacertideformus* positive by Sanger sequencing) were included in the assay. If oral swabs collected at the completion of antibiotic treatment were qPCR negative, the head, liver and/or diseased tissue fixed in alcohol from that animal were tested to confirm the accuracy of the negative result. A gecko was defined as qPCR positive for *E. lacertideformus* when one or more of the samples returned a positive result.

#### Histopathology

The head and body of the formalin-fixed remains (n = 45) were sectioned mid-sagittally. Tissues were decalcified for 20 h (Richard-Allan Scientific Decalcifying Solution, ThermoFisher Scientific), paraffin-embedded, sectioned at 4 μm, and stained with Haematoxylin and Eosin. Tissues were examined microscopically to (1) confirm the presence or absence of *E. lacertideformus*, (2) confirm that lesions were not caused by a non-target pathogen, and (3) determine the efficacy of administered antibiotics as assessed by the extent of *E. lacertideformus* infection and the immune response elicited. The severity of each lesion was scored from 0 to 4 (the percentage replacement of normal tissue volume); where 0 = no lesion present, 1 = a mild lesion (1–25%), 2 = a moderate lesion (26–50%), 3 = a severe lesion (51–75%), and 4 = an extensive lesion (76–100%).

**Figure 1:**
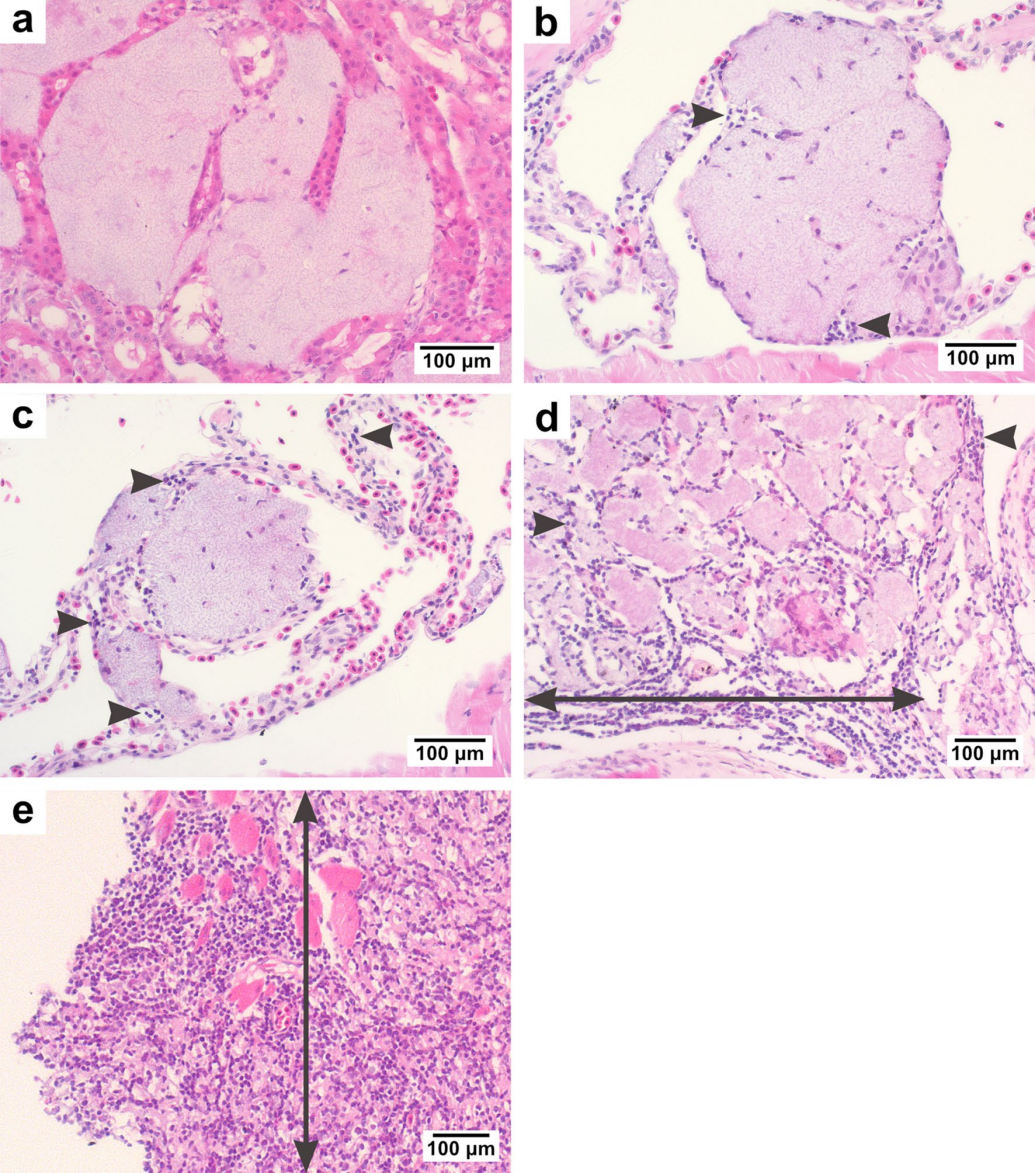
Histological sections stained with Haematoxylin and Eosin showing the range of inflammatory severity in Asian house geckos infected with *E. lacertideformus*. (**a**) Coalescing colonies of *E. lacertideformus* within the kidney associated with no inflammation (inflammatory score = 0), (**b**) large focal colony of *E. lacertideformus* in the lung containing mild inflammation adjacent to the bacterial colonies with perilesional cuffing (inflammatory score = 1, grey arrowheads), (**c**) focal colony of *E. lacertideformus* in the liver containing moderate inflammation adjacent to the bacterial colonies with perilesional cuffing and minimal inflammatory infiltrate (inflammatory score = 2, grey arrowheads), (**d**) multifocal colonies of *E. lacertideformus* markedly distending the soft tissues of the mandible and containing multifocal to coalescing inflammatory infiltrate with evidence of fibroplasia (inflammatory score = 3, double pointed arrow demonstrates location and range of inflammation), (**e**) dispersed colonies of *E. lacertideformus* replacing the soft tissue of the buccal subcutis or lamina propria and containing extensive and diffuse inflammatory infiltrates with evidence of fibroplasia (inflammatory score = 4, grey arrowheads, double pointed arrow demonstrates the location and range of inflammation).

The host inflammatory response was scored on a scale from 0 to 4 (Fig. 1); where 0 = no inflammation (Fig. 1a), 1 = mild inflammation adjacent to the bacterial colonies +/− perilesional cuffing and/or inflammatory infiltrate (Fig. 1b), 2 = moderate inflammation adjacent to the bacterial colonies +/− perilesional cuffing and/or inflammatory infiltrate (Fig. 1c), 3 = multifocal inflammatory infiltration into the lesion +/− evidence of fibroplasia (Fig. 1d), 4 = extensive and diffuse inflammatory infiltration into the lesion +/− evidence of fibroplasia (Fig. 1e). For each gecko, the total lesion score was calculated for all anatomical regions and grouped by the respective antibiotic treatment (including animals not treated with antibiotics). A boxplot was generated to compare the severity of histological lesions across different treatments using the pandas library^13^ v.1.2.0 in Python v3.8^11^.

A non-parametric Kruskal–Wallis test^17^ was used to investigate if there were differences in the tissue inflammatory response in *Hemidactylus frenatus* based on the type of treatment (including no antibiotic treatment) administered. The antibiotic treatments included enrofloxacin (n = 4), amoxicillin-clavulanic acid (n = 4), the combination therapy (n = 3), clarithromycin (n = 4), rifampicin (n = 4), and the no antibiotic treatment group (n = 8). A post-hoc pairwise Tukey honest significant (HSD) comparisons with ‘Bonferroni’ correction was undertaken to compare differences between antibiotics. The package ‘dunn.test’ was used to undertake post-hoc analyses^18^. All analyses were undertaken in the statistical program R^19^. An inflammatory reaction of score ≥ 3 was interpreted to indicate an appropriate host immune response, classifying the antibiotic as more effective. Inflammatory scores below this indicated a poor or ineffective immune response. Differences were considered statistically significant with p ≤ 0.05.

## Results

### qPCR optimisation, specificity and LOD

The qPCR primers and probe targeting a 171 bp fragment of *E. lacertideformus* were successfully developed and optimised. The optimal final primer and probe concentrations were 600 and 250 nM, respectively. Amplification did not occur in the NTCs or when DNA from non-target bacterial species was tested.

A gBlock dilution equivalent to the detection of 122 copies/μL was determined to be the limit of detection. The average Cq value for the LOD corresponded to a Cq and standard deviation of 36.95 ± 0.16 (COV = 0.45), respectively, with no Cq values produced in any of the replicates at dilution levels below this. The reaction efficiency (*E*) and regression coefficient (*R*^*2*^) values were 92.39 and 0.99, respectively.

### Confirmation of *Enterococcus lacertideformus* infection status and its clinical course

All geckos assigned to the experimental inoculation groups (including co-housing treatment), and the control group prior to experimental challenge were confirmed negative for *E. lacertideformus* via qPCR of oral and skin swabs. All three geckos used for the collection of infected tissue for experimental inoculation were confirmed positive for *E. lacertideformus* by qPCR. Both affected geckos collected for the infection by contact trial were confirmed positive via qPCR in the samples collected prior to co-housing. The infection status of Asian house geckos experimentally inoculated with *E. lacertideformus* were confirmed by qPCR of oral and FNA swabs in animals grossly positive for *E. lacertideformus* (displaying lesions visible to the naked eye), and via qPCR of oral swabs and tissue samples, and histology in animals grossly negative for *E. lacertideformus* (Table 3). Prior to enlistment into the treatment trial, all geckos confirmed grossly infected with *E. lacertideformus* had a positive oral swab and/or FNA swab by qPCR (Table 3). All naturally infected geckos used in the treatment trial were positive for *E. lacertideformus* via qPCR. No evidence of additional pathogens were detected via any of the diagnostic means in geckos infected with *E. lacertideformus*.

**Table 3:** Prevalence of gross lesions and infection status of Asian house geckos experimentally challenged with *E. lacertideformus* by six different inoculation methods.

Treatment group	Gross disease status^†^		Gross disease positive				Gross disease negative					
			Disease status—qPCR*				Disease status—qPCR*				Histology	
			Oral swab		FNA lesion		Oral swab		Tissue		Tissue	
	+ (%)	− (%)	+ (%)	− (%)	+ (%)	− (%)	+ (%)	− (%)	+ (%)	− (%)	+ (%)	− (%)
Oral cavity	2 (40%)	3 (60%)	2 (100%)	0 (0%)	2 (100%)	0 (0%)	0 (0%)	3 (100%)	0 (0%)	3 (100%)	0 (0%)	3 (100%)
Subcutaneous	0 (0%)	5 (100%)	n/a	n/a	n/a	n/a	0 (0%)	5 (100%)	3 (60%)	2 (40%)	3 (60%)	2 (40%)
Mucosal abrasion	3 (60%) ‡	2 (40%)	3 (100%)	0 (0%)	3 (100%)	0 (0%)	2 (100%)	0 (0%)	2 (100%)	0 (0%)	1 (50%)	1 (50%)
Skin laceration	5 (100%)	0 (0%)	1 (20%)	4 (80%)	5 (100%)	0 (0%)	n/a	n/a	n/a	n/a	n/a	n/a
Coelomic cavity	3 (60%) ‡	2 (40%)	0 (0%)	3 (100%)	3 (100%)	0 (0%)	0 (0%)	2 (100%)	0 (0%)	2 (100%)	0 (0%)	2 (100%)
Co-housing	0 (0%)	5 (100%)	n/a	n/a	n/a	n/a	2 (40%)	3 (60%)	2 (40%)	3 (60%)	0 (0%)	5 (100%)

The type of diagnostic method used (qPCR or histology), and the type of sample tested by each method (oral swab, FNA lesion, or tissue) based on the gross disease status of the gecko are displayed in the headers of the table.

All remaining geckos confirmed grossly positive for *E. lacertideformus* were enlisted into the antibiotic treatment trial.

+, positive for *E. lacertideformus*; −, negative for *E. lacertideformus.*

^†^A gecko was defined as grossly positive for *E. lacertideformus* when an FNA cytology of the lesion demonstrated characteristic organisms.

*A gecko was defined as qPCR positive for *E. lacertideformus* when the oral swab and/or FNA of the gross lesion tested returned a positive result.

^‡^A gecko grossly positive for *E. lacertideformus* infection but not included in the antibiotic treatment trial (mucosal abrasion, n = 3; coelomic cavity, n = 1).

**Figure 2:**
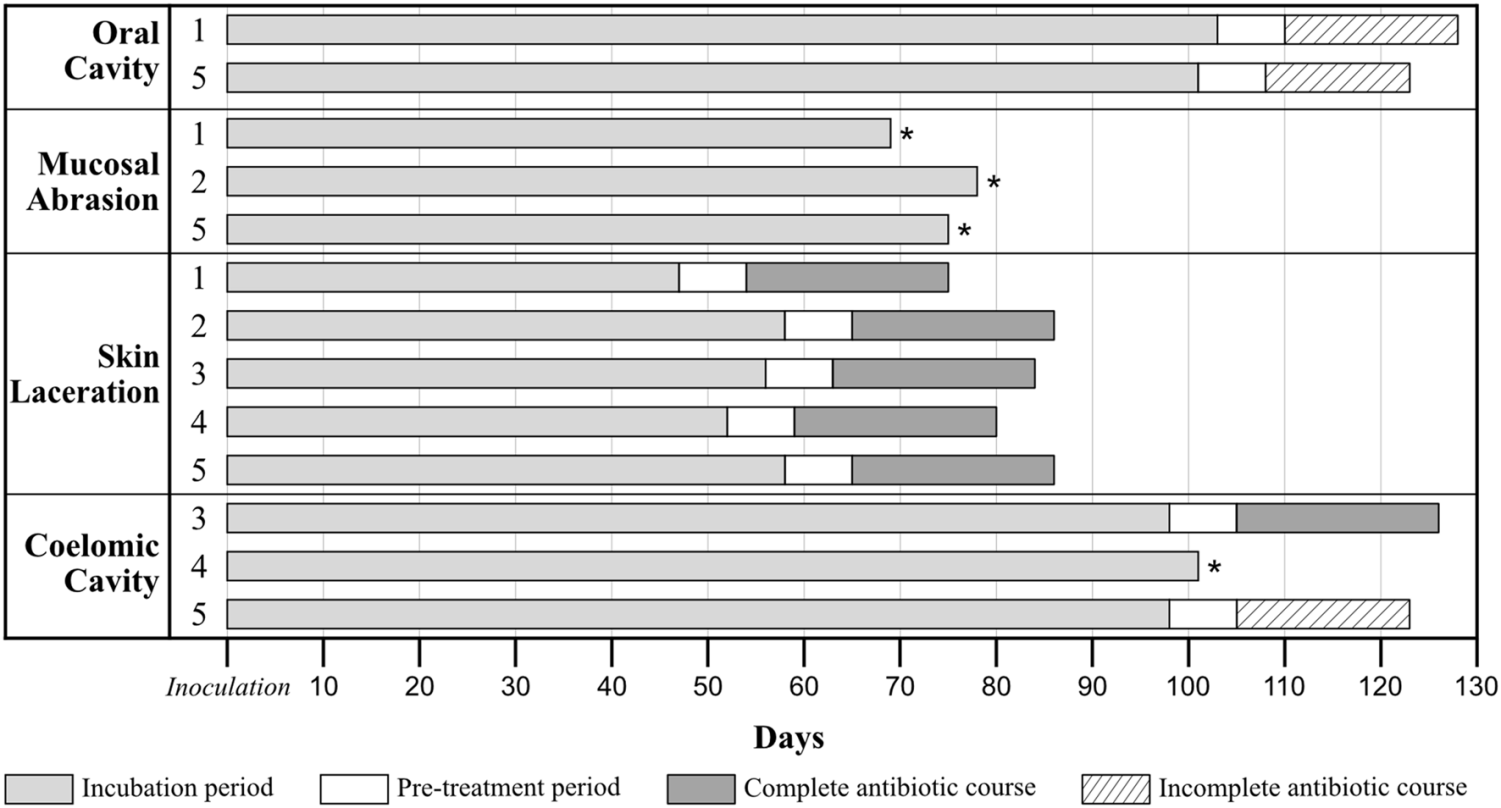
Bar chart illustrating the incubation period, pre-treatment period (period from confirmation of infection and onset of treatment), and treatment duration as a function of route of *E. lacertideformus* inoculation. White, light grey, dark grey, and hatch represent the pre-treatment period, incubation period, complete antibiotic course, and incomplete antibiotic course, respectively. * indicates a gecko that did not receive antibiotic treatment due to logistical limitations. Note this diagram does not include geckos in the subcutaneous inoculation group (SuC) and naturally infected geckos (NaI) as they did not develop clinical disease signs.

The clinical course of experimentally and naturally infected geckos is summarised in Supplementary Table S1. Of the animals that developed disease, geckos infected via the skin laceration and mucosal abrasion routes had the shortest incubation periods, averaging 54 (47 to 58 days, n = 5) and 74 (69 to 78 days, n = 3) days, respectively. Geckos inoculated via coelomic injection and by mouth averaged longer incubation periods of 99 (98 to 101 days, n = 3) and 102 (101 to 103 days, n = 2) days, respectively. Geckos in the subcutaneous injection and co-housing groups did not develop gross lesions (Fig. 2).

### Macroscopic lesions of geckos infected with *E. lacertideformus*

**Figure 3:**
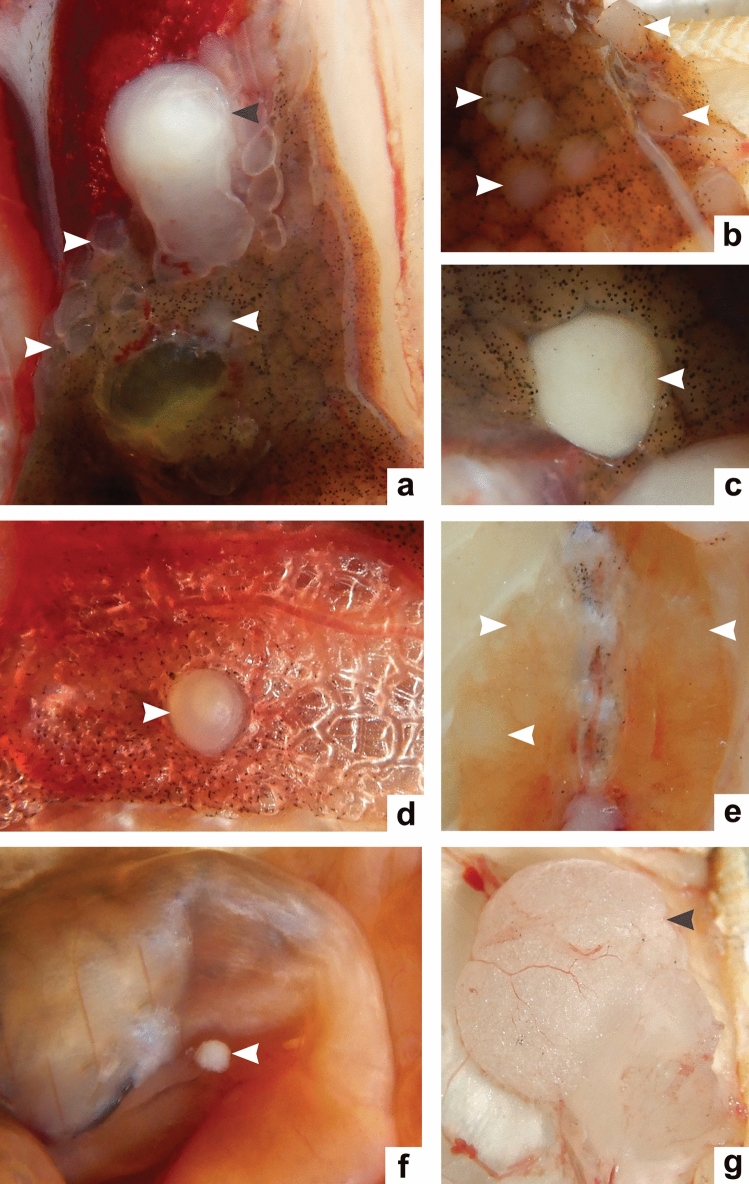
Macroscopic findings of Asian house geckos infected with *E. lacertideformus*. One large raised white focus adherent to the surface of the lung (grey arrowhead) and multiple smaller foci extending from the surface of the liver (white arrowheads) (gecko ID: CoC-4) (**a**), multifocal masses extensively replacing the hepatic parenchyma (white arrowheads) (gecko ID: SkL-2) (**b**), replacement of the gallbladder lumen with bacterial colonies (white arrowhead) (gecko ID: SuC-3) (**c**), a single raised white focus replacing parenchyma of the left lung and distending the pleura (white arrowhead) (gecko ID: NaI-9) (**d**), bilateral replacement of renal parenchyma by multiple raised white foci (white arrowheads) (gecko ID: SkL-2) (**e**), single raised white focus adhered to serosa of the lesser curvature of the stomach (white arrowhead) (gecko ID: SkL-2) (**f**), single large ventral mass cranial to pelvis (grey arrowhead) (gecko ID: CoC-3) (**g**).

No macroscopic lesions were seen in geckos infected with *E. lacertideformus* through the subcutaneous route. When geckos were inoculated by the skin laceration route, lesions developed at the site of inoculation (cheek) in three of five geckos, and in two of five geckos’ lesions developed in the cheek and maxillary mucosa or perivascular tissues surrounding the eye (Supplementary Table S2). When the inoculum was administered by mouth, gross lesions in the oral mucosa of the mandible or maxilla developed in two of five geckos. Abrasion of the oral mucosa resulted in lesions in three of five geckos in the mandibular mucosa. The coelomic cavity injection resulted in a single ventral abdominal mass in three of five geckos. The skin laceration route was the only inoculation method to produce macroscopic lesions in all geckos. The distribution of the macroscopic lesions observed in the viscera of the infected geckos is shown in Fig. 3. Gross bacterial colonies were found in the kidney (n = 9), liver (n = 6), pancreas (n = 3), lung (n = 2), stomach (n = 1), and colon (n = 1) of *E. lacertideformus* infected geckos (Supplementary Table S2).

### Change in the surface area of macroscopic lesions following treatment

**Figure 4:**
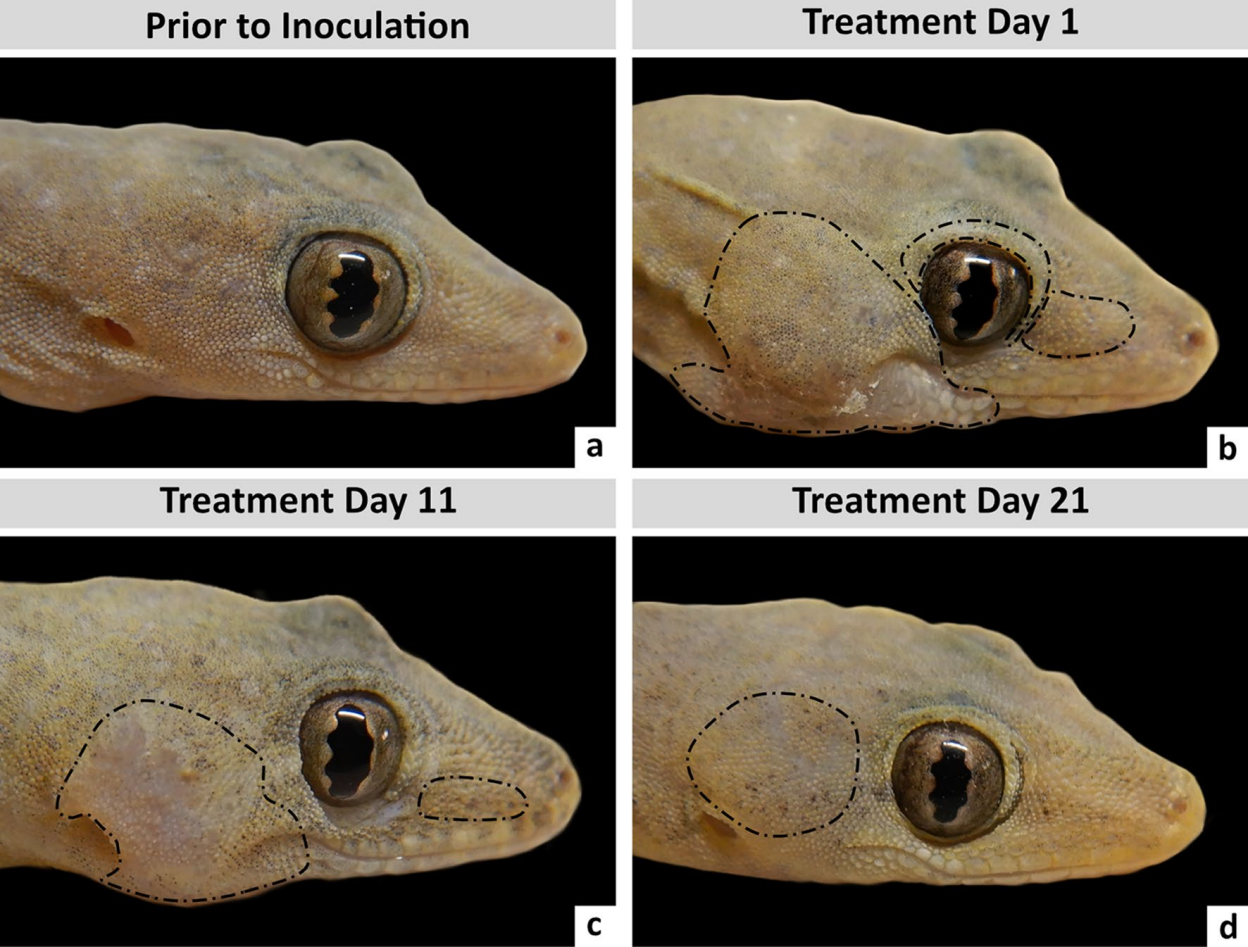
Treatment timeline of an Asian house gecko (gecko ID: SkL-4) experimentally infected with *E. lacertideformus* where lesions are delimited with a broken black line to illustrate the near complete regression of gross disease following treatment with enrofloxacin*.* Gecko prior to experimental inoculation via skin laceration at the right cheek (**a**). Representative lesions of *E. lacertideformus* observed at one (**b**), eleven (**c**), and 21 days (**d**) of oral antibiotic treatment with enrofloxacin. Near complete regression of gross lesions during antibiotic treatment is apparent, with markedly reduced subcutaneous swelling of the right cheek caudal to the eyes and cranial to the ear canal ostium (**b**–**d**).

**Figure 5:**
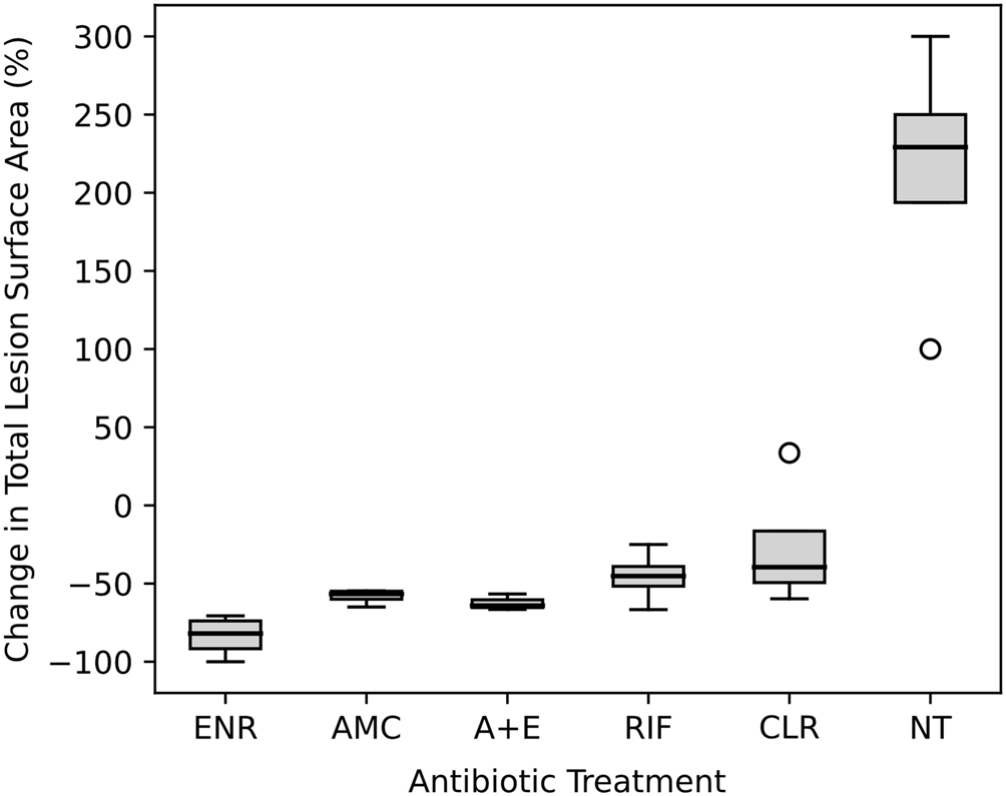
Boxplot of the average percentage change in total lesion surface area for each antibiotic treatment administered. The severity of each lesion was scored from 0 to 4 (the percentage replacement of normal tissue volume); where 0 = no lesion present, 1 = a mild lesion (1–25%), 2 = a moderate lesion (26–50%), 3 = a severe lesion (51–75%), and 4 = an extensive lesion (76–100%). The bold line indicates the median, the interquartile range (25th to 75th percentile) is represented by the grey shading, the whiskers represent the minimum and maximum values, and the outlier is shown by the circles. *ENR* enrofloxacin; *AMC* amoxicillin-clavulanic acid; *A + E* amoxicillin-clavulanic acid, and enrofloxacin; *RIF* rifampicin; *CLR* clarithromycin; *NT* no treatment.

Geckos given enrofloxacin (n = 4), amoxicillin-clavulanic acid (n = 4), and the combination therapy (n = 3) had the greatest reduction in lesion surface area over the treatment period (Fig. 4), ranging from 54.7 to 100.0% (Fig. 5). When geckos were given clarithromycin (n = 4) and rifampicin (n = 4), the reduction in the lesion surface area ranged from 25.0 to 66.7%, except for a single gecko given clarithromycin which had an increase in lesion surface area by 33.3% (Fig. 5). The lesion surface area in geckos given enrofloxacin, the combination therapy, amoxicillin-clavulanic acid, rifampicin, and clarithromycin changed by an average of − 83.6 (range: − 100.0 to − 70.7), − 62.4 (range: − 66.6 to − 56.7), − 58.2 (range: − 65.0 to − 54.7), − 45.5 (range: − 66.7 to − 25.0), and − 26.5 (range: − 60.0 to + 33.3)%, respectively (Supplementary Table S2).

Three geckos inoculated by mucosal abrasion (n = 3) and one inoculated by coelomic cavity injection (n = 1) were not included in the treatment trial due to a delay in identifying the onset of gross disease. Macroscopic lesions of experimentally infected geckos not treated with antibiotics continued to enlarge until they were euthanised at day 28 (Supplementary Table S2). Over the disease course, the lesion surface area at the inoculation site in untreated geckos within the mucosal abrasion group increased between 233.3 and 300.0%. The lesion surface area of the single untreated gecko in the coelomic cavity group increased 100.0%.

### Distribution of lesions

**Figure 6:**
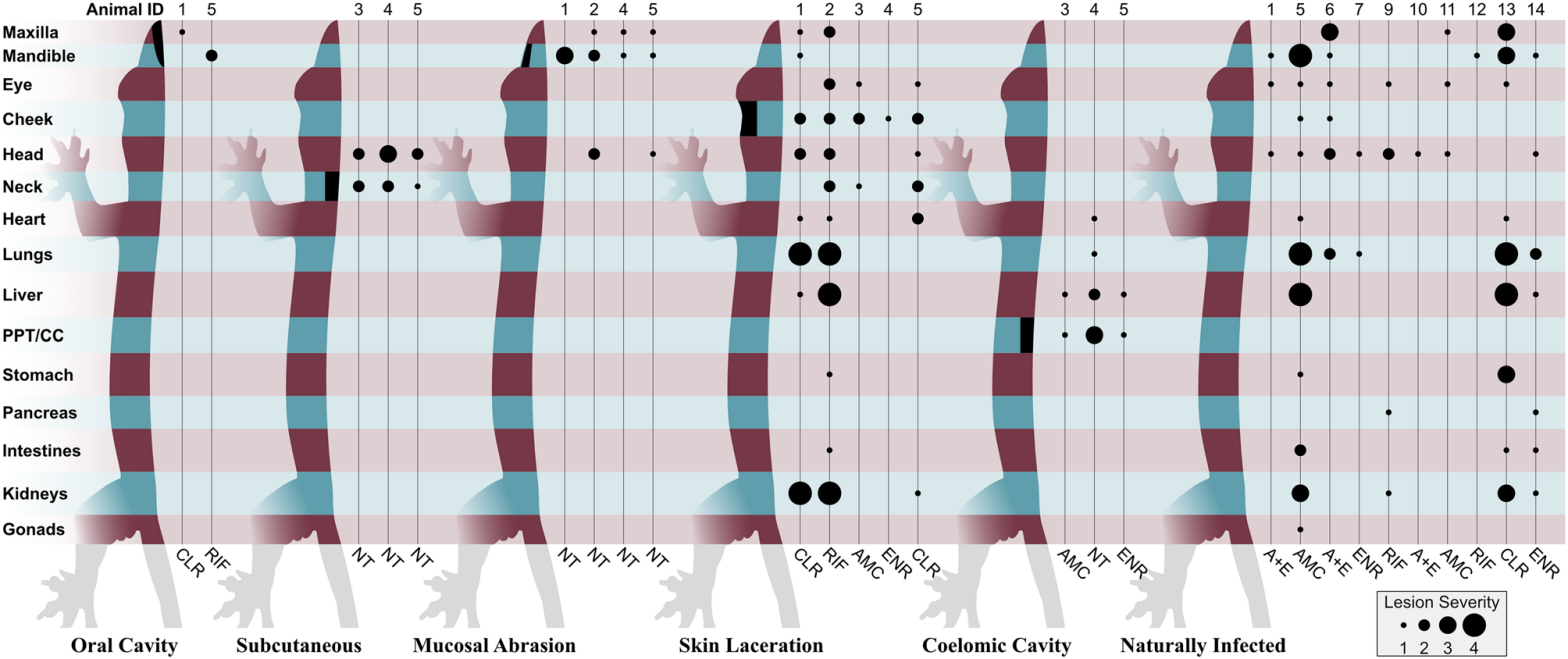
Histological distribution of *E. lacertideformus* in experimentally infected Asian house geckos per inoculation route and treatment group. The numerical values positioned at the top of the figure represent the ID of each infected gecko. The treatment administered to each gecko are abbreviated; *AMC* amoxicillin-clavulanic acid; *A + E* amoxicillin-clavulanic acid and enrofloxacin; *CLR* clarithromycin; *ENR* enrofloxacin; *NT* no treatment; *RIF* rifampicin. PPT/CC represents lesions associated with the pleural and pericardial tissues and/or the coelomic cavity. The section of black shading on the gecko represents the site of inoculation for that group. Identification of *E. lacertideformus* bacteria in organs/tissues are represented by the black circles. The severity of the histological lesions from 1 to 4 are denoted by the size of the circle.

Evidence of *E. lacertideformus* infection was observed in the tissues of 17 experimentally infected geckos (Supplementary Table S3). Irrespective of the antibiotic treatment status, geckos inoculated via the skin laceration route were observed to have a more extensive disease spread from the original inoculation site (Fig. 6). Lesions in geckos inoculated via the oral cavity, mucosal abrasion, and subcutaneous injection were confined to the head and neck (Fig. 6). Dissemination of *E. lacertideformus* in geckos inoculated via the coelomic route and treated with antibiotics were confined to the liver, pericardium, pleura, and injection site (coelomic cavity), however, the bacteria were observed to have disseminated to the parenchyma of the lungs and heart in one gecko not treated with antibiotics. The severity of the bacterial lesions across all groups was most pronounced in the skin laceration group, in which the highest disease grade (grade 4) was documented in two of the five inoculated geckos (Fig. 6).

Evidence of *E. lacertideformus* was observed in the tissues of ten geckos infected naturally (Supplementary Table S3). All naturally infected geckos had histological lesions in one or more parts of the head, particularly at the crown, within the mandible, and the region surrounding the eyes. Most of the naturally infected geckos had dissemination of *E. lacertideformus* organisms to internal organs/tissues (60%, n = 6), particularly to the lungs (50%, n = 5) and kidney (40%, n = 4). Irrespective of the antibiotic, geckos with advanced disease (i.e. spread to multiple internal organs/tissues) typically had higher lesion severity scores than geckos with disease confined entirely to the region of the head (Fig. 6).

### Histological inflammatory response

**Figure 7:**
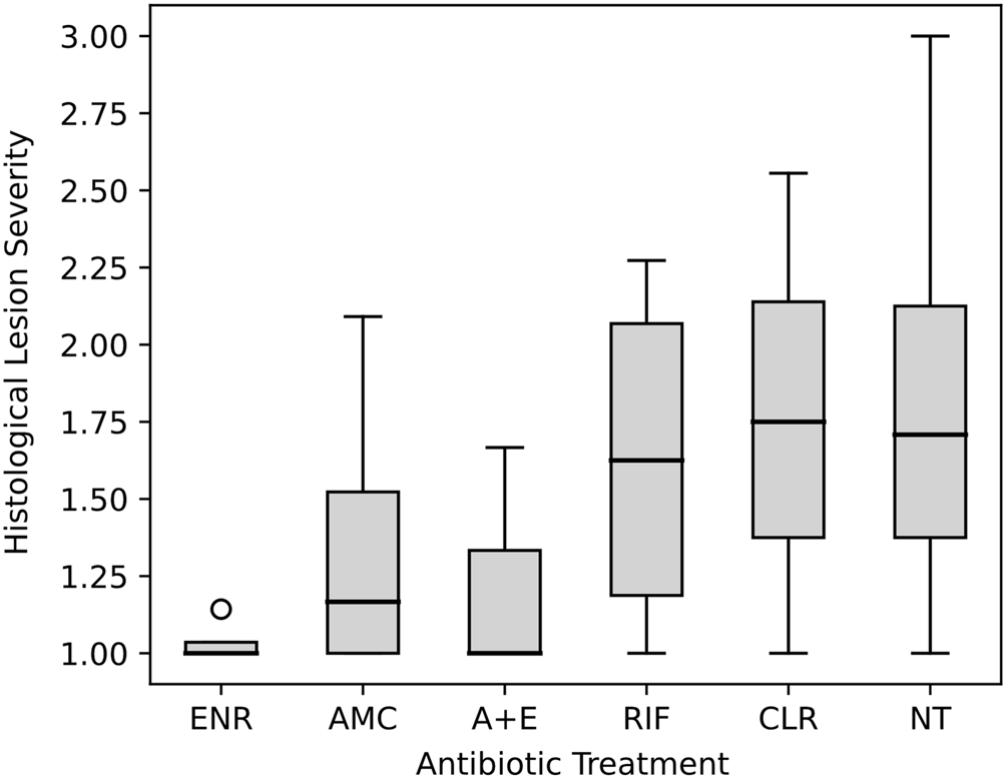
Boxplot of the average histological lesion severity for each antibiotic treatment administered. The bold line indicates the median, the interquartile range (25th to 75th percentile) is represented by the grey shading, the whiskers represent the minimum and maximum values, and the outlier is shown by the circle. *ENR* enrofloxacin; *AMC* amoxicillin-clavulanic acid; *A + E* amoxicillin-clavulanic acid and enrofloxacin; *RIF* rifampicin; *CLR* clarithromycin; *NT* no treatment.

Histologically, if inflammation was present, the predominant leukocyte associated with *E. lacertideformus* were lymphocytes (Fig. 1; Supplementary Table S4). Scattered heterophils admixed with the lymphocytes were common, and histiocytes and multinucleate giant cells were observed less frequently. An inflammatory response to *E. lacertideformus* was absent in five of eight geckos that were not treated, the remaining two geckos had a mild inflammatory response (Inflammation score = 1), and a single gecko had a moderate inflammatory response (Inflammation score ranging from 2 to 3). A mild inflammatory response was observed in all geckos administered clarithromycin and rifampicin (Fig. 7), however, a single gecko given rifampicin had a mild to moderate inflammatory response (Inflammation score ranging from 2 to 3). In experimentally and naturally infected geckos, the most pronounced inflammatory responses (Inflammation scores ranging from 3 to 4), predominantly characterised by infiltration of immune cells, destruction of bacterial colonies, and fibroplasia, were associated with geckos administered enrofloxacin, amoxicillin-clavulanic acid, and the combination therapy (Fig. 7; Supplementary Table S4).

The Kruskal–Wallis test revealed there was a significant difference of antibiotic treatment on the histological inflammatory score observed in Asian house geckos (X^2^ = 18.45, df = 5, p = 0.002). The pairwise Tukey honest significant comparisons with ‘Bonferroni’ correction post-hoc analyses identified that there was a significant difference between enrofloxacin and no treatment (individuals that were infected with *E. lacertideformus*, but not treated with antibiotics) (p = 0.007), and there was a significant difference between amoxicillin clavulanic acid and no treatment (p = 0.016). Additionally, there was a statistical tendency between the combined treatment and no treatment (p = 0.136). There were no significant differences between other antibiotic treatments and between antibiotic treatments and no treatments (Table 4) (Supplementary Table S5).

**Table 4:** Results from Tukey’s honest significant tests (HSD). Significant differences are bolded at the 95% confidence interval.

Post-hoc comparisons	Z score	P value
A + E—AMC	− 0.36116	1
A + E—CLR	1.529635	0.945
AMC—CLR	2.042296	0.308
A + E—ENR	− 0.55237	1
AMC—ENR	− 0.20652	1
CLR—ENR	− 2.24882	0.183
A + E—NT	2.3608	0.136
AMC—NT	3.060413	**0.016**
CLR—NT	0.702173	1
ENR—NT	3.298887	**0.007**
A + E—RIF	1.50839	0.985
AMC—RIF	2.019349	0.325
CLR—RIF	− 0.02295	1
ENR—RIF	2.225873	0.195

*ENR* enrofloxacin; *AMC* amoxicillin-clavulanic acid; *A* + *E* amoxicillin-clavulanic acid and enrofloxacin; *RIF* rifampicin; *CLR* clarithromycin; *NT* no treatment.

### Confirmation of *Enterococcus lacertideformus* infection status at euthanasia

The infection status of all treated Asian house geckos at treatment completion or euthanasia was confirmed via qPCR of oral swabs and/or tissues, and histology (Table 5). Following 21 days of treatment, all geckos were confirmed infected with *E. lacertideformus* by histology and qPCR (Table 5). At trial completion all members of the non-inoculated control group were negative for *E. lacertideformus* via all detection means. Both naturally affected geckos caught for the co-housing trial were positive for *E. lacertideformus* via qPCR at euthanasia.

**Table 5:** Infection status of Asian house geckos given antibiotics at the end of the treatment period.

Treatment group	Disease status		Disease status—qPCR*				Histology^†^	
	Gross		Oral swab		Tissue			
	+ (%)	− (%)	+ (%)	− (%)	+ (%)	− (%)	+ (%)	− (%)
Oral cavity	2 (100%)	0 (0%)	2 (100%)	0 (0%)	2 (100%)	0 (0%)	2 (100%)	0 (0%)
Skin laceration	5 (100%)	0 (0%)	1 (20%)	4 (80%)	5 (100%)	0 (0%)	5 (100%)	0 (0%)
Coelomic cavity	2 (100%)	0 (0%)	0 (0%)	2 (100%)	2 (100%)	0 (0%)	2 (100%)	0 (0%)
Naturally infected	9 (90%)	1 (10%)	10	0 (0%)	10 (100%)	0 (0%)	10 (100%)	0 (0%)

*A gecko was defined as qPCR positive for *E. lacertideformus* when the oral swab and/or tissue samples returned a positive result.

^†^A gecko was defined as histologically positive for *E. lacertideformus* when characteristic organisms were observed in the tissues.

## Discussion

The first objective of this study was to determine if Asian house geckos could be experimentally infected with *E. lacertideformus*. This study showed that disease closely resembling that seen in geckos naturally infected with *E. lacertideformus* can be experimentally induced by application of an *E. lacertideformus* suspension to lacerated skin or abraded oral mucosa, by subcutaneous injection, and by oral administration. Intraperitoneal injection with *E. lacertideformus* was also able to induce an infection, however, the lesions were confined to the coelomic cavity. Although only small numbers of animals were used in this study, it appears that inoculating a skin laceration will most consistently result in infection and disease, and the time between inoculation and the onset of gross lesions will be shortest. Also, this was the only route of inoculation that resulted in dissemination from the original inoculation site, most similar to natural infection. This study additionally showed that infection can be transmitted between geckos by co-housing them, although, only oral colonisation was demonstrated, and disease did not develop over the course of the trial.

These findings provide insight into the potential mechanisms of *E. lacertideformus* transmission between infected and uninfected reptiles. Asian house geckos and blue-tailed skinks are known to bite each other during fighting and mating^20,21^, and cannibalism in Asian house geckos has been documented^21^. Biting also occurs as male blue-tailed skinks subdue other male blue-tailed skinks in same sex sexual encounters. In both these instances, if the biting lizard had an oral infection with *E. lacertideformus* and is able to penetrate the skin of the lizard they are biting, then this natural behaviour effectively replicates the skin laceration inoculation group in this study. Cannibalism of an infected gecko by an uninfected gecko could also potentially result in infection if the bacteria were released from a head or a coelomic lesion when the animal was being consumed.

How *E. lacertideformus* is transmitted between the co-housed geckos in this study is not known. No evidence of aggression, including bite wounds, was observed during the co-housing period, however, geckos were not monitored continuously, and therefore, aggression may have occurred. Another possible route of transmission would be that of environmental contamination by the infected gecko. Both geckos likely drank water from the same source. Also, while not yet studied, based on previous histological evidence of oral and gastrointestinal lesions shedding organisms, it is possible that *E. lacertideformus* could be shed in faeces resulting in faecal-oral contamination. To confirm these potential routes of transmission, water and faecal samples should be aseptically collected and subjected to the *E. lacertideformus*-specific qPCR*.* Both geckos infected during the co-housing trial had oral colonisation by *E. lacertideformus* but did not develop disease during the experimental period. This finding is consistent with the observation that the geckos experimentally infected with the oral inoculation route took the longest to develop disease.

The second objective of this study was to determine if antibiotics could be used successfully to cure infected geckos. The treatments administered during the trial were chosen based on one or more of the following criteria: efficacy against Gram positive bacteria, ability to penetrate biofilms, therapeutic index, previous use in reptiles, capacity to administer via the oral route, and susceptibility to *E. lacertideformus* and other species of enterococci. A screen of the *E. lacertideformus* genome for antimicrobial resistance genes in a previous study revealed a resistance profile only to the antibiotics trimethoprim, tetracycline, streptothricin, and bacitracin^7^.

All five treatment protocols used in this study caused a reduction in the size of gross lesions. However, it appears that geckos treated with enrofloxacin had the largest reduction in the size of their gross lesions, the lowest average lesion scores, and were more likely to have lesions containing an appropriate inflammatory response, possibly in response to dying bacteria. Although enrofloxacin shows promise, only a small sample size of geckos were used, and this antibiotic is considered a last-resort antibacterial for the treatment and prevention of infections in humans^22^. However, enrofloxacin is also extensively administered in veterinary medicine as a result of its therapeutic properties and practicality in administration^23,24^, and thus, should not be eliminated as a potential treatment option for *E. lacertideformus*. Amoxicillin-clavulanic acid also showed a similar impact on *E. lacertideformus* induced lesions and would be a potential option for treatment. Combination therapy using both enrofloxacin and amoxicillin-clavulanic acid did not appear to improve the treatment outcome.

Despite evidence of the bactericidal activity and biofilm penetrating capability of enrofloxacin^25^ and amoxicillin-clavulanic acid treatments^26,27^, all but one gecko, an enrofloxacin treated animal, were shown to remain grossly infected with *E. lacertideformus* at the end of the 21-day treatment period. Therefore, it is unclear whether antimicrobial treatment will completely clear an animal of infection with longer treatment periods, or treatment periods with an increased dose or dosage frequency. The majority of geckos treated in this study continued to eat and maintain their weight during the treatment period, so a longer duration of treatment could appear to be a safe option. Single-dose oral administration of enrofloxacin in Asian house geckos has been shown to reach concentrations that exceed the minimum inhibitory concentrations that would be effective for enterococcal species when given at the dosage rate (10 mg/kg) used in this study^28^. However, as *E. lacertideformus* produces a biofilm, and biofilm producing bacteria often require an antimicrobial MIC up to 100 times higher than planktonic bacteria^29,30^, the dosage rate and frequency would likely need to be increased. Increasing the dosage rate or frequency would lead to increased blood and likely tissue concentrations of the antibiotic, and these increased concentrations might be more effective at penetrating the biofilm and achieving the therapeutic range needed to eliminate *E. lacertideformus.* However, this would not be recommended unless additional pharmacokinetic studies were undertaken to ensure that drug concentrations did not become toxic.

## Conclusion

The Asian house gecko represents a foundational model to study the dynamics of *E. lacertideformus* disease. This gecko is a highly invasive species of similar mass, physiology, and preferred habitat to the critically endangered Lister's gecko and blue-tailed skink on Christmas Island. Active monitoring of *E. lacertideformus* infected reptiles*,* and the development of treatment protocols proves imperative as multisystemic spread of the organism and ultimate death have been documented in all cases of untreated lizards^2^. This research showed that infection of geckos with *E. lacertideformus* closely resembling that seen in geckos infected naturally can be established experimentally via inoculation, and naturally by means of direct contact through co-housing. However, inoculation of a skin wound appears to result in the highest infection rate, with the shortest incubation period, and greatest chance of dissemination to the coelomic viscera, most closely mimicking natural infection. Due to frequent colonisation of the oral cavity by *E. lacertideformus* in experimentally and naturally infected geckos, the molecular analysis of oral swabs collected from wild or captively housed reptiles using the developed *E. lacertideformus*-specific qPCR may serve as a non-invasive and reliable diagnostic tool and disease surveillance method. When geckos infected with *E. lacertideformus* were treated with antibiotics all geckos exhibited some degree of lesion regression, though cure was not achieved in any case. *Enterococcus lacertideformus* appears to be sensitive to enrofloxacin, and amoxicillin-clavulanic acid, *in-vitro*, but treatment regiments more than 21 days may be required to achieve a cure at the dosage rates administered in this study. Further research into the pharmacology of amoxicillin-clavulanic acid, and enrofloxacin in Asian house geckos, particularly those naturally infected will be necessary to define the appropriate dose, dose frequency, and period of treatment required to achieve the therapeutic range needed to eliminate *E. lacertideformus*. Knowledge of the drugs MIC may lead to the development of novel and highly effective therapeutic and prophylactic treatment protocols necessary to protect susceptible reptiles.

## Supplementary Information

Supplementary Information.

## Data Availability

Most data generated and analysed during this study are included in this published article (and its Supplementary Information files). Other datasets are available from the corresponding author on reasonable request.
