# Supplementary material for: Experimental infection of Asian house geckos with Enterococcus lacertideformus demonstrates multiple disease transmission routes and the in-vivo efficacy of antibiotics
Source: Sci Rep. 2021 Jul 5;11:13858. doi: 10.1038/s41598-021-92999-y (PMC8257746; doi:10.1038/s41598-021-92999-y)
Supplement: Supplementary file 1 — Supplementary Information. [file 41598_2021_92999_MOESM1_ESM.docx]

**Supplementary Table 1:** Signalment and clinical course of infection with *E. lacertideformus* in Asian house geckos.

| **Lizard ID** | **Sex** | **Day Signs Were First Observed** | **Antibiotic** | **Day of Euthanasia** | **Days of Antibiotic Treatment or Disease** | **Condition at Euthanasia✝** |
| --- | --- | --- | --- | --- | --- | --- |
| OrC-1 | F | 103 | CLR | 128 | 18 | Moribund |
| OrC-5 | M | 101 | RIF | 123 | 15 | Moribund |
| SuC-3* | F | NCS | NT | 140 | n/a | Well |
| SuC-4* | M | NCS | NT | 144 | n/a | Well |
| SuC-5* | M | NCS | NT | 112 | n/a | Well |
| MuA-1* | F | 69 | NT | 97 | 28** | Well |
| MuA-2* | F | 78 | NT | 106 | 28** | Well |
| MuA-3* | M | NCS | NT | 120 | n/a | Stable |
| MuA-4* | M | NCS | NT | 120 | n/a | Well |
| MuA-5* | M | 75 | NT | 103 | 28** | Well |
| SkL-1 | F | 47 | CLR | 75 | 21 | Well |
| SkL-2 | F | 58 | RIF | 86 | 21 | Well |
| SkL-3 | M | 56 | AMC | 84 | 21 | Well |
| SkL-4 | M | 52 | ENR | 80 | 21 | Well |
| SkL-5 | M | 58 | CLR | 86 | 21 | Well |
| CoC-3 | F | 98 | AMC | 126 | 21 | Well |
| CoC-4* | M | 101 | NT | 129 | 28** | Well |
| CoC-5 | M | 98 | ENR | 123 | 18 | Moribund |
| CoH-2* | F | NCS | NT | 120 | n/a | Well |
| CoH-5* | M | NCS | NT | 120 | n/a | Well |
| NaI-1 | F | n/a | A+E | 21 | 21 | Stable |
| NaI-5 | M | n/a | AMC | 21 | 21 | Stable |
| NaI-6 | M | n/a | A+E | 21 | 21 | Stable |
| NaI-7 | F | n/a | ENR | 21 | 21 | Well |
| NaI-9 | M | n/a | RIF | 21 | 21 | Well |
| NaI-10 | F | n/a | A+E | 21 | 21 | Well |
| NaI-11 | M | n/a | AMC | 21 | 21 | Well |
| NaI-12 | F | n/a | RIF | 21 | 21 | Well |
| NaI-13 | F | n/a | CLR | 21 | 21 | Well |
| NaI-14 | M | n/a | ENR | 21 | 21 | Well |

CoC, Coelomic cavity; CoH, Co-housing; MuA, Mucosal abrasion; NaI, Naturally infected; OrC, Oral cavity; SkL, Skin laceration; SuC, Subcutaneous injection; A+E, Amoxicillin-clavulanic acid and enrofloxacin; AMC, Amoxicillin-clavulanic acid; CLR, Clarithromycin; ENR, Enrofloxacin; NI, Naturally infected; NT, No treatment; RIF, Rifampicin; NCS, No clinical signs observed.

*, Animal positive in disease (either via qPCR, histology, and/or cytology) that did not receive treatment. **, Days of observable *E. lacertideformus* disease in an animal that did not receive antibiotic treatment.
✝ - Well, gecko that was alert and observed to be exhibiting natural behaviours, with a consistent appetite and/or faeces production; Stable, gecko that was alert, with a reduced appetite, and/or faeces production; Moribund, gecko exhibiting lethargy, with a cessation in appetite, and/or faeces production.

**Supplementary Table 2:** Macroscopic lesions of Asian house geckos with *E. lacertideformus* infection at the start of treatment and at euthanasia.

| **Lizard ID** | **Antibiotics** | **Description of CS‡** | **Gross Clinical Signs at Euthanasia** | **Change in Lesion Size (%)** | **Gross Internal Lesions✝** |
| --- | --- | --- | --- | --- | --- |
| OrC-1 | CLR | Swelling of R maxillary mucosa (2.5 x 1 mm) extending 40% from the commissure to the midline. | Anorexia and lethargy. Swelling of R maxillary mucosa (2 x 0.5 mm). | -60.0% | Appeared normal. |
| OrC-5 | RIF | Swelling of L mandibular mucosa (5 x 1.5 mm) extending 70% from the commissure to midline. | Anorexia and lethargy. Swelling of L mandibular mucosa (4 x 1 mm). | -46.7% | Appeared normal. |
| SuC-3* | NT | n/a | NCS | n/a | Opaque and cloudy gallbladder enlarged pancreas. |
| SuC-4* | NT | n/a | NCS | n/a | Appeared normal. |
| SuC-5* | NT | n/a | NCS | n/a | Enlarged R kidney containing small multifocal white masses, and pale pancreas. |
| MuA-1* | NT | Swelling of R mandibular mucosa (4.5 x 1 mm) extending 40% from the commissure to midline. | Swelling of R mandibular mucosa (6 x 3 mm) extending 20% from the commissure to midline, with ulceration and minimal petechiae. | 300.0% | Gallbladder with cloudy contents, bilateral hypertrophy of proximal kidneys with whitened fatty appearance. |
| MuA-2* | NT | Swelling of R mandibular mucosa (3 x 0.5 mm) extending 25% from the commissure to midline. | Swelling of R mandibular mucosa (5 x 1 mm) extending from the commissure to midline. Rostral SC swelling cranial to eyes (3 x 4 mm). | 233.3% | White streaking deposits on kidney. |
| MuA-3* | NT | n/a | NCS | n/a | Appeared normal. |
| MuA-4* | NT | n/a | NCS | n/a | Distended large intestine. |
| MuA-5* | NT | Swelling of R rostral mandibular mucosa extending to L (4 x 1 mm). | Swelling of R rostral mandibular mucosa extending to L (6.5 x 2 mm), most prominent at the rostrum. Mucosal epithelium reddened with petechiae. | 225.0% | Enlarged pancreas containing small to medium multifocal white masses. |
| SkL-1 | CLR | SC swelling of R cheek caudal to eye and cranial to ear canal ostium (6 x 5 mm). | Swelling of R cheek (5 x 4 mm), marginally more flattened. Rostral SC swelling cranial to eyes (4 x 6 mm). | -33.3% | Pale liver. Miliary small white foci liver and R kidney. |
| SkL-2 | RIF | SC swelling of R cheek rostro-ventrally to ear canal ostium (4 x 4 mm) and extending to and surrounding perivascular tissues of the eye (3.5 mm thickness). Swelling of R maxillary mucosa (3 x 1 mm) extending 30% from the commissure to midline. | Swelling of R cheek (3 x 3mm) and extending to R eye (2 mm thickness). Swelling of R maxillary mucosa (2 x 0.5 mm) | -43.8% | Distended and cloudy gallbladder, enlarged liver with extensive multifocal white masses, enlarged L kidney, bilateral small to medium renal parenchymal white masses. Small, focal gastric serosal mass. |
| SkL-3 | AMC | Swelling of R cheek extending ventrally from the eye to the ear canal ostium (5 x 6 mm). | Swelling of R cheek (3 x 3.5 mm). Swelling moderately flattened. | -65.0% | Small focal mass in pancreas. |
| SkL-4 | ENR | SC swelling of R cheek extending from the eye to the ear canal ostium (6 x 5 mm). | Swelling of R cheek (3 x 2.5 mm), extensively more flattened. | -75.0% | Cloudy gallbladder. |
| SkL-5 | CLR | SC swelling of R cheek extending ventrally from the eye to the ear canal ostium (4 x 3 mm) and surrounding the eye (2 mm thickness). Diffuse pale purple bruising on crown of head and encircling R eye. | Swelling of R cheek (4 x 4 mm) and R eye (1 mm thickness), marginally more flattened. Diffuse light yellow/brown bruising on crown of head and eye. | 33.3% | Small mass on R kidney. |
| CoC-3 | AMC | Firm ventral mass (5 x 4 mm) adjacent to the midline and cranial to the L pelvic inlet. | Ventral mass (3 x 3 mm), moderately more flattened. Epithelium reddened with petechiae. | -55.0% | White soft tissue nodule (6 x 4 mm) L of midline in line with hip. |
| CoC-4* | NT | Firm ventral mass (4 x 3 mm) adjacent to the midline and cranial to the L pelvic inlet. | Ventral mass (6 x 4 mm), slightly more raised. | 100.0% | Cloudy gallbladder, large focal white mass adhered to lung surface (5 x 3 mm) surrounded by less opaque small to medium multifocal masses extending from the proximal to distal portion of the liver. |
| CoC-5 | ENR | Firm ventral mass (3 x 3 mm) adjacent to midline and caudal to the edge of L liver lobe. | Lethargy. Ventral mass (1 x 1 mm), extensively more flattened. | -88.9% | Enlarged R liver lobe. |
| CoH-2* | NT | n/a | NCS | n/a | Appeared normal. |
| CoH-5* | NT | n/a | NCS | n/a | Appeared normal. |
| NaI-1 | A+E | Large mass of R mandibular mucosa (9 x 6 mm) extending from R commissure to L of the rostral midline resulting in ventral displacement. Mucosal surfaces ulcerated with petechiae. Multifocal raised SC epithelial lesions of lower labials and extending to chin shields (4 x 9 mm), more prominent on the R. | Anorexia and lethargy. Swelling of R mandibular mucosa (6 x 3.5mm) with no ulceration and minimal petechiae. Multifocal lower labial lesions (3 x 6 mm), moderately more flattened. | -55.6% | Distended and cloudy gallbladder. |
| NaI-5 | AMC | Large mass of L mandibular mucosa (7 x 4 mm) extending from L commissure to R of the midline resulting in ventral displacement. Mucosal surfaces ulcerated with petechiae. Multifocal raised SC epithelial lesions of lower labials and extending to chin shields (5 x 3 mm), more prominent on the L. | Moderate lethargy. Swelling of L mandibular mucosa (5 x 3 mm) with no ulceration and minimal petechiae. Multifocal lower labial lesions (3 x 1.5 mm), moderately more flattened. | -58.2% | Cloudy gallbladder, enlarged liver (approximately double the normal size), and extensive small multifocal masses replacing entire liver. |
| NaI-6 | A+E | Swelling of L rostral mandibular and maxillary mucosa (4 x 2.5mm), crossing the midline and extending 70% of the R mandible and maxilla (7 x 3.5 mm). Multi-focal to coalescing SC nodular masses on the crown of the head, neck, and frontal (ranging: 5 x 1 – 6 x 5 mm). Rostral SC swelling cranial to eyes (4 x 6 mm). | Swelling of L mandibular and maxillary mucosa (2.5 x 1.5 mm) and extending to the R (4 x 1.5mm). Multi-focal epithelial lesions on crown of head, neck and frontal (3 x 1 – 4 x 3mm), moderately more flattened. Rostral swelling cranial to eyes (2.5 x 4.5 mm). | -63.7% | Appeared normal. |
| NaI-7 | ENR | Multifocal raised SC epithelial lesions of R lower labial scales and extending to chin shields (4 x 3 mm). Multifocal firm SC nodular masses (1 x 1 – 2.5 x 2 mm) on dorsal aspect extending from the shoulders to pelvis. | No evidence of epithelial lesions or other signs of disease. | Complete lesion regression | Distended and cloudy gallbladder. |
| NaI-9 | RIF | SC swelling at medial crown of head (5 x 6 mm) caudal to eyes. | Swelling at crown of head (5 x 4.5 mm), minimally more flattened. | -25.0% | Single cloudy mass on R lung, single white mass on colon, enlarged pancreas containing multifocal small white masses, and small multifocal clear masses in R kidney. |
| NaI-10 | A+E | SC swelling medial crown of head (6 x 8mm) extending to the rostrum (7 x 5mm). | Swelling at crown of head (3.5 x 4.5 mm), moderately more flattened, and rostrum (4 x 3 mm). | -66.5% | Distended and cloudy gallbladder. |
| NaI-11 | AMC | SC swelling cranial to the eyes and extending to the tip of the rostrum (6 x 8 mm). | Rostral swelling (4 x 5 mm), extensively more flattened. | -58.3% | Enlarged L kidney with multifocal large white masses. |
| NaI-12 | RIF | L swelling caudo-ventrally to eye (4 x 3 mm). | L swelling (2 x 2 mm), moderately more flattened. | -66.7% | Appeared normal. |
| NaI-13 | CLR | SC swelling of the inter-mandibular space (8 x 6 mm), crown of head (5 x 5 mm), and L ventral eye (3.5 x 1.5 mm). Swelling of L mandibular mucosa (3.5 x 1 mm) extending 60% from the commissure to the midline. | Swelling of inter-mandibular space (6 x 4.5 mm). Swelling at crown of head (3 x 3 mm), moderately flattened. Swelling of L ventral eye (4 x 1.5 mm). Swelling of L mandibular mucosa (2 x 1 mm). | -34.1% | Cloudy gallbladder, enlarged liver with large white multifocal to coalescing masses replacing entire organ, pale kidneys with bilateral hypertrophy and a whitened fatty appearance. |
| NaI-14 | ENR | SC swelling medial crown of head (6 x 5 mm) extending to the rostrum (4 x 4 mm). | Swelling at crown of head (3 x mm). Rostral swelling (3 x 2.5 mm), extensively more flattened. | -66.6% | Appeared normal. |

CoC, Coelomic cavity; CoH, Co-housing; MuA, Mucosal abrasion; NaI, Naturally infected; OrC, Oral cavity; SkL, Skin laceration; SuC, Subcutaneous injection; NCS; No clinical signs observed; L, Left; R, Right.

‡, Description of day 7 clinical signs in geckos untreated and treated with antibiotics.

✝, Gross internal lesions consistent with *E. lacertideformus* infection.

*, Geckos not treated with antibiotics.

**Supplementary Table 3:** Scoring of histological lesions per tissue/organ in Asian house geckos experimentally and naturally infected with *E. lacertideformus*.

| Lizard ID | Antibiotics | Maxilla | Mandible | Eye | Cheek | Head‡ | Neck | Heart | Lungs | Liver | PPT/CC | Stomach | Pancreas | Intestines | Kidney | Gonads |
| --- | --- | --- | --- | --- | --- | --- | --- | --- | --- | --- | --- | --- | --- | --- | --- | --- |
| OrC-1 | CLR | 1 | – | – | – | – |  | – | – | – | – | – | * | – | – | – |
| OrC-5 | RIF | – | 2 | – | – | – |  | – | – | – | – | – | * | – | – | – |
| SuC-3 | NT | – | – | – | – | 2 | 2 | – | – | – | – | – | – | – | – | * |
| SuC-4 | NT | – | – | – | – | 3 | 2 | – | – | – | – | – | – | – | – | – |
| SuC-5 | NT | – | – | – | – | 2 | 1 | – | – | – | – | – | – | – | – | – |
| MuA-1 | NT | – | 3 | – | – | – | – | – | – | – | – | – | * | – | – | – |
| MuA-2 | NT | 1 | 2 | – | – | 2 | – | – | – | – | – | – | * | – | * | * |
| MuA-4 | NT | 1 | 1 | – | – | – | – | – | – | – | – | – | * | – | – | – |
| MuA-5 | NT | 1 | 1 | – | – | 1 | – | – | – | – | – | – | * | – | * | – |
| SkL-1 | CLR | 1 | 1 | – | 2 | 2 | – | 1 | 4 | 1 | – | – | * | – | 4 | – |
| SkL-2 | RIF | 2 | – | 2 | 2 | 2 | 2 | 1 | 4 | 4 | – | 1 | * | 1 | 4 | – |
| SkL-3 | AMC | – | – | 1 | 2 | – | 1 | – | – | – | – | – | * | – | – | * |
| SkL-4 | ENR | – | – | – | 1 | – | – | – | – | – | – | – | * | – | – | – |
| SkL-5 | CLR | – | – | 1 | 2 | 1 | 2 | 2 | – | – | – | – | * | – | 1 | – |
| CoC-3 | AMC | – | – | – | – | – | – | – | – | 1 | 1 | – | – | – | – | – |
| CoC-4 | NT | – | – | – | – | – | – | 1 | 1 | 2 | 3 | – | * | – | – | – |
| CoC-5 | ENR | – | – | – | – | – | – | – | – | 1 | 1 | – | – | – | – | – |
| NaI-1 | A+E | – | 1 | 1 | – | 1 | – | – | – | – | – | – | * | – | – | * |
| NaI-5 | AMC | – | 4 | 1 | 1 | 1 | – | 1 | 4 | 4 | – | 1 | * | 2 | 3 | 1 |
| NaI-6 | A+E | 3 | 1 | 1 | 1 | 2 | – | * | 2 | – | – | – | – | – | – | – |
| NaI-7 | ENR | – | – | – | – | 1 | – | * | 1 | – | – | – | * | – | – | – |
| NaI-9 | RIF | – | – | 1 | – | 2 | – | – | – | – | – | – | 1 | – | 1 | – |
| NaI-10 | A+E | – | – | – | – | 1 | – | – | – | – | – | – | * | – | – | – |
| NaI-11 | AMC | 1 | – | 1 | – | 1 | – | – | – | – | – | – | – | – | – | * |
| NaI-12 | RIF | – | 1 | – | – | – | – | – | – | – | – | – | * | – | – | * |
| NaI-13 | CLR | 3 | 3 | 1 | – | – | – | 1 | 4 | 4 | – | 3 | – | 1 | 3 | * |
| NaI-14 | ENR | – | 1 | – | – | 1 | – | – | 2 | 1 | – | – | 1 | 1 | 1 | – |

CoC, Coelomic cavity; CoH, Co-housing; MuA, Mucosal abrasion; NaI, Naturally infected; OrC, Oral cavity; SkL, Skin laceration; SuC, Subcutaneous injection; A+E, Amoxicillin-clavulanic acid and enrofloxacin; AMC, Amoxicillin-clavulanic acid; CLR, Clarithromycin; ENR, Enrofloxacin; PPT/CC, Pleural pericardial tissues/coelomic cavity; NT, No treatment; RIF, Rifampicin.

‡, Indicates crown of head.

*, Indicates missing organ/tissue in histology section.

Lesions were scored on a scale of 0 to 4; where 0 = no lesion present (normal), 1 = a mild lesion, 2 = a moderate lesion, 3 = a severe lesion, 4 = an extensive lesion.

Data points marked with ‘–’ indicate that no *E. lacertideformus* organisms were detected.

**Supplementary Table 4:** Histological grading of inflammation in Asian house geckos experimentally and naturally infected with *E. lacertideformus*.

| **Lizard ID** | **Abs** | **Inflammation score** | **Description of inflammatory response** |
| --- | --- | --- | --- |
| OrC-1 | CLR | 1 | Lymphocytic infiltrate. |
| OrC-5 | RIF | 1 | Scattered heterophils admixed with lymphocytes. |
| SuC-3 | NT | 2-3 | Scattered multinucleate giant cells in lymphocytic infiltrate. |
| SuC-4 | NT | 0 | n/a |
| SuC-5 | NT | 0 | n/a |
| MuA-1 | NT | 1 | Lymphocytic infiltrate. |
| MuA-2 | NT | 0 | n/a |
| MuA-4 | NT | 0 | n/a |
| MuA-5 | NT | 0 | n/a |
| SkL-1 | CLR | 1 | Scattered heterophils admixed with lymphocytic infiltrate. |
| SkL-2 | RIF | 1 | Scattered heterophils admixed with lymphocytic infiltrate. |
| SkL-3 | AMC | 3-4 | Scattered heterophils admixed with lymphocytic infiltrate. Loss of bacterial definition and evidence of cell death (absent chains, retention of biofilm and CT replacement of bacterial colonies). |
| SkL-4 | ENR | 3-4 | Scattered multinucleate giant cells in lymphocytic infiltrate. Loss of bacterial definition and evidence of cell death (absent chains, retention of biofilm and CT replacement of bacterial colonies). |
| SkL-5 | CLR | 1 | Inflammatory infiltrate comprising approximately 30% heterophils and 70% lymphocytes. |
| CoC-3 | AMC | 2-3 | Scattered heterophils admixed with lymphocytic infiltrate. Early evidence of fibroplasia. |
| CoC-4 | NT | 1 | Scattered heterophils admixed with lymphocytic infiltrate. |
| CoC-5 | ENR | 2-3 | Early evidence of fibroplasia. |
| NaI-1 | A+E | 2-3 | Inflammatory infiltrate comprising approximately 40% heterophils and 60% lymphocytes. Early evidence of fibroplasia. |
| NaI-5 | AMC | 2 | Inflammatory infiltrate comprising approximately 40% heterophils and 60% lymphocytes. |
| NaI-6 | A+E | 2-3 | Scattered heterophils admixed with lymphocytic infiltrate. Loss of bacterial definition and evidence of cell death (absent chains, retention of biofilm and CT replacement of bacterial colonies). |
| NaI-7 | ENR | 2-3 | Scattered histiocytes admixed with lymphocytic infiltrate. Early evidence of fibroplasia. |
| NaI-9 | RIF | 0-1 | Scattered heterophils admixed with lymphocytes. |
| NaI-10 | A+E | 1-2 | Scattered heterophils admixed with lymphocytic infiltrate. |
| NaI-11 | AMC | 2-3 | Scattered heterophils admixed with lymphocytic infiltrate. Loss of bacterial definition and evidence of cell death (absent chains, retention of biofilm and CT replacement of bacterial colonies). |
| NaI-12 | RIF | 1-2 | Scattered heterophils admixed with lymphocytic infiltrate. |
| NaI-13 | CLR | 1 | Scattered heterophils admixed with lymphocytic infiltrate. |
| NaI-14 | ENR | 2-3 | Scattered heterophils admixed with lymphocytic infiltrate. Early evidence of fibroplasia. |

CoC, Coelomic cavity; CoH, Co-housing; MuA, Mucosal abrasion; NaI, Naturally infected; OrC, Oral cavity; SkL, Skin laceration; SuC, Subcutaneous injection; CT, Connective tissue. A+E, Amoxicillin-clavulanic acid and enrofloxacin; AMC, Amoxicillin-clavulanic acid; CLR, Clarithromycin; ENR, Enrofloxacin; NT, No treatment; RIF, Rifampicin.

Inflammation was scored on a multi-step scale of 0 to 4; where 0 = no inflammation, 1 = mild inflammation adjacent to the bacterial colonies +/- perilesional cuffing and/or inflammatory infiltrate, 2 = moderate inflammation adjacent to the bacterial colonies +/- perilesional cuffing and/or inflammatory infiltrate, 3 = multifocal inflammatory infiltration into the lesion +/- evidence of fibroplasia, 4 = extensive and diffuse inflammatory infiltration into the lesion +/- evidence of fibroplasia.

**Supplementary Table 5:** The model mean, standard deviation, standard error, and 95% confidence interval of the inflammation scores for each antibiotic treatment.

| **Antibiotic** | **No. of individuals** | **Mean score** | **SD** | **SE** | **95% CI** |
| --- | --- | --- | --- | --- | --- |
| A+E | 3 | 2.167 | 0.58 | 0.33 | 1.43 |
| AMC | 4 | 2.63 | 0.63 | 0.31 | 1.00 |
| CLR | 4 | 1 | 0.00 | 0.00 | 0.00 |
| ENR | 4 | 2.75 | 0.50 | 0.25 | 0.80 |
| NT | 8 | 0.56 | 0.90 | 0.32 | 0.76 |
| RIF | 4 | 1.00 | 0.41 | 0.20 | 0.65 |
